# Estimated Testing, Tracing, and Vaccination Targets for Containment of the US Mpox Outbreak

**DOI:** 10.1001/jamanetworkopen.2022.50984

**Published:** 2023-01-13

**Authors:** Melanie H. Chitwood, Jiye Kwon, Alexandra Savinkina, Jo Walker, Alyssa Bilinski, Gregg Gonsalves

**Affiliations:** 1 Department of Epidemiology of Microbial Diseases, Yale School of Public Health, New Haven, Connecticut; 2 Public Health Modeling Unit, Yale School of Public Health, New Haven, Connecticut; 3 Department of Health Services, Policy, and Practice, Brown School of Public Health, Providence, Rhode Island; 4 Department of Biostatistics, Brown School of Public Health, Providence, Rhode Island

## Abstract

This decision analytic model estimates the levels of community testing, contract tracing, and vaccination required to reduce the effective reproduction number of the mpox virus among the high-risk group of men who have sex with men.

## Introduction

As of November 2022, there have been more than 28 000 confirmed US mpox cases, predominantly among men who have sex with men (MSM).^1^ Reducing transmission of mpox virus (hMPXV) is crucial to protect the health of MSM and reduce the risk of transmission in the general population. While testing, contact tracing, and vaccination can slow the spread of hMPXV, target levels of these measures have not been established. We aimed to estimate the levels of community testing, contact tracing, and vaccination required to reduce the effective reproduction number (R_t_) of hMPXV to less than 1 for high-risk MSM (HR-MSM).

## Methods

In this decision analytic model, we adapted a deterministic branching model to describe the transmission of hMPXV among HR-MSM^2^ (eMethods and eFigure in Supplement 1). Infectious individuals are detected depending on the community detection rate and whether their source case underwent contact tracing (eFigure in Supplement 1). We assumed that community detection of a case reduced secondary infections by 50%, identifying a case early through contact tracing reduced secondary infections by 90%, and all detected cases have the same probability of being contact traced (eTables 1 and 2 in Supplement 1). We found the minimum level of vaccine coverage that would reduce R_t_ to less than 1, accounting for contact tracing and community detection. To reflect uncertainty in the basic reproduction number (R_0_) in the HR-MSM population, we varied R_0_ from 1.2 to 2.0.^3^

Analyses were conducted using R version 4.2.1 (R Project for Statistical Computing). This study does not qualify as human participant research; no institutional review board approval was sought, and informed consent was not required. The analysis follows relevant Consolidated Health Economic Evaluation Reporting Standards (CHEERS) reporting guidelines.

## Results

**Figure 1.**
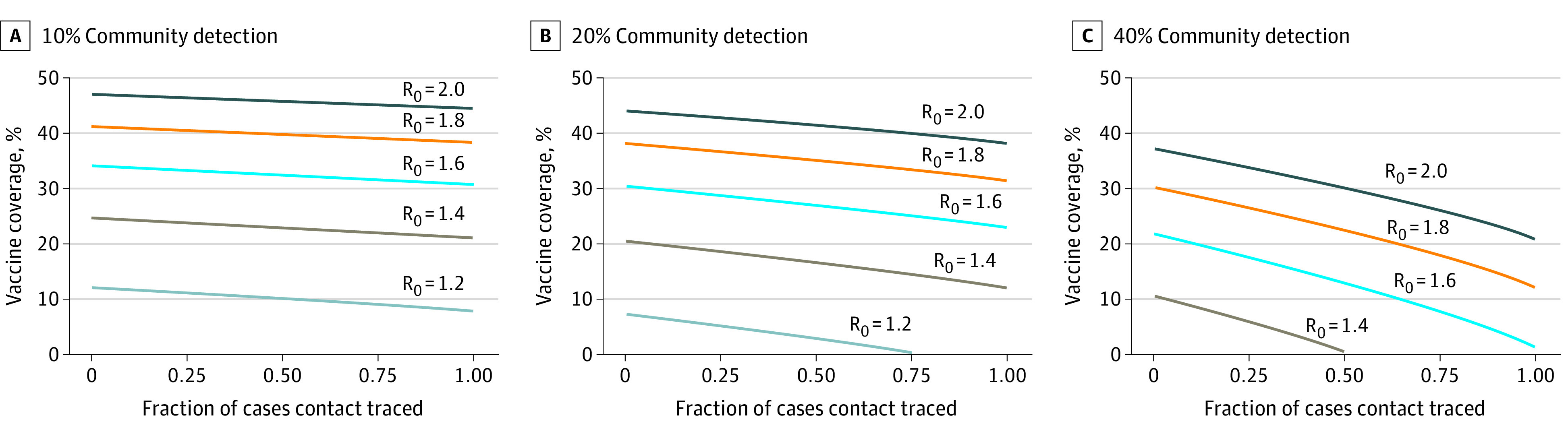
Frontier of Contact Tracing, Testing, and Vaccination Coverage to Reduce the Effective Reproduction Number to Less Than 1 The community detection rate varied across panels. Each line shows the efficient frontier for a given value of the basic reproduction number (R_0_). Each point on the line can be interpreted as the minimum vaccination coverage at which the effective reproduction number remains less than 1 for a given value of R_0_ and contacts traced.

We found that testing and contact tracing without vaccination can reduce R_t_ to less than 1 among HR-MSM if the R_0_ is less than 1.4, at least 40% of cases are detected through community testing, and at least 50% of contacts are traced. With a moderate response (ie, ≥20% community detection; ≥25% of cases contact traced) the critical threshold to vaccinate ranges from 5% (R_0_ = 1.2) to 43% (R_0_ = 2.0) (Figure 1). In this scenario, 170 000 to 1.4 million full doses of the Modified Vaccinia Ankara-Bavarian Nordic vaccine (Bavarian Nordic) would need to be administered to 85 000 to 731 000 of the 1.7 million MSM who are eligible for preexposure prophylaxis (a proxy for HR-MSM) in the United States.^4,5^

**Figure 2.**
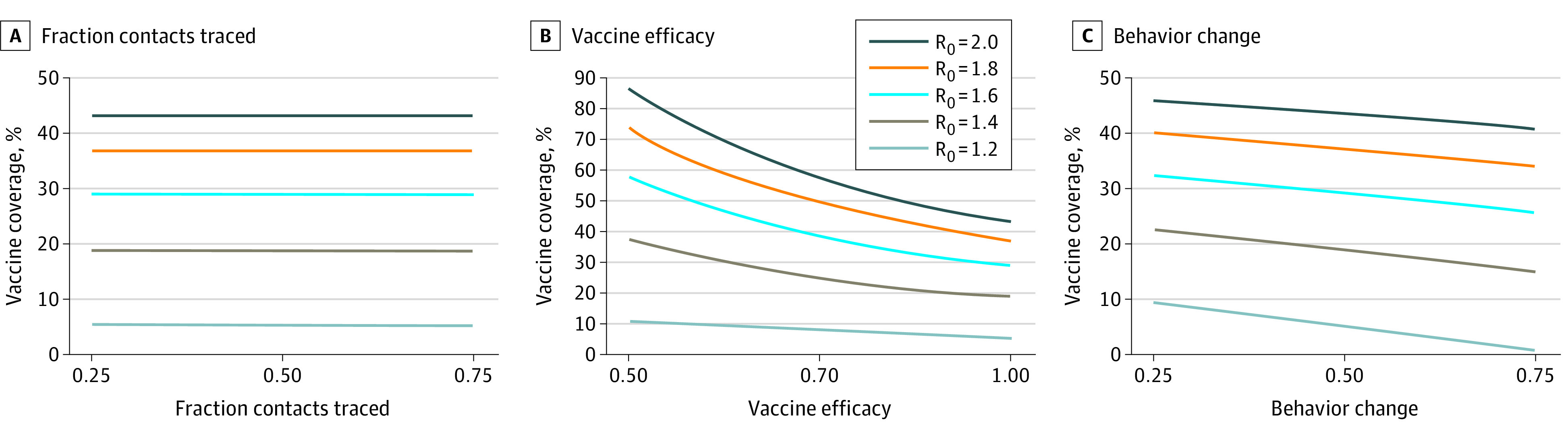
Association of Key Model Parameters With the Critical Threshold to Vaccinate All panels assume a community detection rate of 20% and 25% of cases are contact traced. Each line shows the efficient frontier, describing the minimum vaccination coverage at which the effective reproduction number remains less than 1 for a given value of the basic reproduction number (R_0_).

The critical threshold to vaccinate was not sensitive to assumptions about the fraction of secondary cases detected through contact tracing (Figure 2A), but it was sensitive to assumptions about vaccine efficacy and the association of self-isolation with secondary infections. For example, if vaccine efficacy were 85%,^6^ the critical threshold to vaccinate ranged from 6% (R_0_ = 1.2) to 51% (R_0_ = 2.0) in the moderate response scenario (Figure 2B). Similarly, as the association between self-isolation and secondary infections decreased, the critical threshold to vaccinate increased (Figure 2C).

## Discussion

In this study, we simulated public health measures to reduce the spread of hMPXV in the United States. We found that the critical threshold to vaccinate depends on the R_0_ of hMPXV and on public health measures. Rapid distribution of vaccinations to at least one-third of the HR-MSM population (at least 1.1 million doses), with increased testing and contact tracing, can support containment efforts in most modeled scenarios. Short-term behavioral changes, such as limiting intimate physical contacts, will support containment efforts but do not obviate the need for vaccination for long-term containment.

This analysis has several limitations. We estimated vaccine requirements assuming that sustained spread outside the HR-MSM population is negligible and vaccines are efficiently targeted to HR-MSM. Consequently, our estimates may represent a lower bound of doses needed. Furthermore, although we illustrated sensitivity of our results to variations in vaccine efficacy and reductions in secondary infections due to behavioral changes, these parameters remain uncertain. Overall, we believe this analysis provides a useful framework for quantitative targets toward hMPXV containment.

## Notes

Supplement 1.
eMethods.
**eTable 1.** Model Parameter Values **eTable 2.** Estimation of Secondary Infections
eReferences.
**eFigure.** Model Schematic

Supplement 2.
Data Sharing Statement
