# Supplementary material for: Estimated Testing, Tracing, and Vaccination Targets for Containment of the US Mpox Outbreak
Source: JAMA Netw Open. 2023 Jan 13;6(1):e2250984. doi: 10.1001/jamanetworkopen.2022.50984 (PMC9857202; doi:10.1001/jamanetworkopen.2022.50984)
Supplement: Supplement 1. — eMethods. eTable 1. Model Parameter Values eTable 2. Estimation of Secondary Infections eReferences. eFigure. Model Schematic [file jamanetwopen-e2250984-s001.pdf]

Supplemental Online Content

Chitwood MH, Kwon J, Savinkina A, Walker J, Bilinski A, Gonsalves G. Estimated testing, tracing, and vaccination targets for containment of the US mpox outbreak. *JAMA Netw Open.* 2023;6(1):e2250984. doi:10.1001/jamanetworkopen.2022.50984

eMethods.

eTable 1. Model Parameter Values

eTable 2. Estimation of Secondary Infections

eReferences.

eFigure. Model Schematic

This supplemental material has been provided by the authors to give readers additional information about their work.

© 2023 Chitwood MH et al. *JAMA Network Open.*

Supplement

Methods

To describe the transmission of monkeypox virus (hMPXV) in a population of high risk men who have sex with men (HR-MSM), we adapted a deterministic branching model first developed for SARS-CoV-2[2]. The model by Bilinski et al. has compartments to account for detection at the asymptomatic, presymptomatic, or symptomatic stage. As transmission of hMPXV is presumed to only occur among symptomatic individuals[7], we removed the asymptomatic and presymptomatic states and transitions (Figure S1). We made additional changes to parameter values to account for the epidemiological differences between the two viruses (Table S1).

We use the model to assess three tools to reduce transmission of hMPXV: community detection, contact tracing, and vaccination. The model estimates the number of secondary infections arising from cases (Table S2). Cases can be detected either through community testing programs or as a result of contact tracing. Community testing identifies individuals once they are infectious; these individuals take measures to reduce their contacts by 50% over the infectious period. Contact tracing identifies individuals prior to the onset of infectiousness; these individuals enter quarantine and reduce their contacts by 90% over the infectious period. Undetected cases do not reduce their contacts and do not undergo contact tracing; the secondary infections arising from undetected cases can be detected through community testing only. All detected cases have the same probability of having their contacts traced. We estimated the rate of vaccination and contact tracing needed to lower R_t below 1, assuming community detection rates of 10%, 20%, and 40%.

Finally, we estimate the number of vaccine doses needed to support containment. Because the Jynneos vaccine requires two doses given at least four weeks apart[8], the number of doses needed is twice the number of individuals that need to be vaccinated. We make the simplifying assumptions that the Jynneos vaccine is 100% efficacious against infection, that protection is conferred immediately following the first dose, that no doses are wasted, and that doses are only given to HR-MSM. As a result, our estimates represent a lower bound on the number of doses needed in each scenario.

© 2023 Chitwood MH et al. *JAMA Network Open.*

eTable 1 . Model Parameter Values

**Parameter**	**Value(s)**	**Source**
Probability of community case detection (k_c)	10%, 20%, 40%	Assumed
Probability of contact tracing case detection (k_t)	50%	Assumed
R0	1.2, 1.4, 1.6, 1.8, 2.0	(4)
Fraction of cases that are contact traced (p)	varies	Assumed
Duration of infectiousness (d)	21 days	(9)
Relative number of secondary infections from detected infections compared to undetected infections (q)<br>• note: this applies to cases that aren't contact traced but are detected through testing, as well as cases that are contact traced (all of which we assume are detected, see above). As a result, the overall transmissibility ratio $\frac{R_{contact\ traced}}{R_{not\ traced\ or\ otherwise\ detected}}$ is computed as the product of this parameter and $(1-\epsilon)$, see below	0.5	Assumed
Average daily rate of transmission for symptomatic cases not traced (b)	calibrated	
Additional reduction in secondary infections due to quarantine (ϵ, only applicable to cases that are contact traced)	80%	Assumed
Vaccination % (v)	Varies	

© 2023 Chitwood MH et al. *JAMA Network Open.*

eTable 2. Estimation of Secondary Infections

Category	Formula
Not contact traced, detected	$r_{ND} = (1-v)bdq$
Not contact traced, undetected	$r_{NU} = (1-v)bd$
Contact traced, detected	$r_{TD} = (1-e)(1-v)bdq$
Contract traced, undetected	$r_{TU} = (1-e)(1-v)bd$

Where:

b= Average daily rate of transmission for cases not traced

d=duration of infectiousness

q=Relative number of secondary infections from detected infections compared to undetected infections

e=Isolation and quarantine efficacy

© 2023 Chitwood MH et al. *JAMA Network Open.*

References

1. 2022 U.S. Map & Case count. Centers for Disease Control and Prevention. https://www.cdc.gov/poxvirus/monkeypox/response/2022/us-map.html. Accessed November 3, 2022. Archived https://archive.ph/88PnZ.
2. Bilinski A, Mostashari F, Salomon JA. Modeling Contact Tracing Strategies for COVID-19 in the Context of Relaxed Physical Distancing Measures. *JAMA Network Open*. 2020;3(8):e2019217-e2019217. doi:10.1001/jamanetworkopen.2020.19217
3. Second meeting of the International Health Regulations (2005) (IHR) emergency committee regarding the multi-country outbreak of Monkeypox. World Health Organization. https://www.who.int/news/item/23-07-2022-second-meeting-of-the-international-health-regulations-(2005)-(ihr)-emergency-committee-regarding-the-multi-country-outbreak-of-monkeypox. Accessed July 28, 2022. Archived https://archive.ph/gPeC4.
4. Neilan AM, Landovitz RJ, Le MH, et al; Cost-Effectiveness of Long-Acting Injectable HIV Preexposure Prophylaxis in the United States: A Cost-Effectiveness Analysis. *Ann Intern Med*. 2022;175:479-489. [Epub 1 February 2022]. doi:10.7326/M21-1548
5. Kimball S. CDC estimates 1.7 million gay and bisexual men face highest risk from monkeypox. CNBC. https://cnb.cx/3QmowsQ Accessed August 21, 2022. Archived https://archive.ph/v7vWl
6. Fine PE, Jezek Z, Grab B, & Dixon H (1988). The transmission potential of monkeypox virus in human populations. *International journal of epidemiology*, *17*(3), 643–650. https://doi.org/10.1093/ije/17.3.643
7. CDC. Monkeypox: How it Spreads [Internet]. Centers for Disease Control and Prevention. Centers for Disease Control and Prevention; 2022 [cited 2022Jul30]. Available from: https://www.cdc.gov/poxvirus/monkeypox/transmission.html. Archived at: https://archive.ph/sQ0Nv.
8. US Food and Drug Administration, Jynneos 125678 package insert, June 21, 2021. [cited 2022Jul30]. Available from: https://www.fda.gov/vaccines-blood-biologics/jynneos. Archived at: https://archive.ph/WwalN.
9. Thornhill, J. P., Barkati, S., Walmsley, S., Rockstroh, J., Antinori, A., Harrison, L. B., Palich, R., Nori, A., Reeves, I., Habibi, M. S., Apea, V., Boesecke, C., Vandekerckhove, L., Yakubovsky, M., Sendagorta, E., Blanco, J. L., Florence, E., Moschese, D., Maltez, F. M., Goorhuis, A., … SHARE-net Clinical Group (2022). Monkeypox Virus Infection in Humans across 16 Countries - April-June 2022. *The New England journal of medicine*, 10.1056/NEJMoa2207323. Advance online publication. https://doi.org/10.1056/NEJMoa2207323

© 2023 Chitwood MH et al. *JAMA Network Open.*

eFigure. Model Schematic

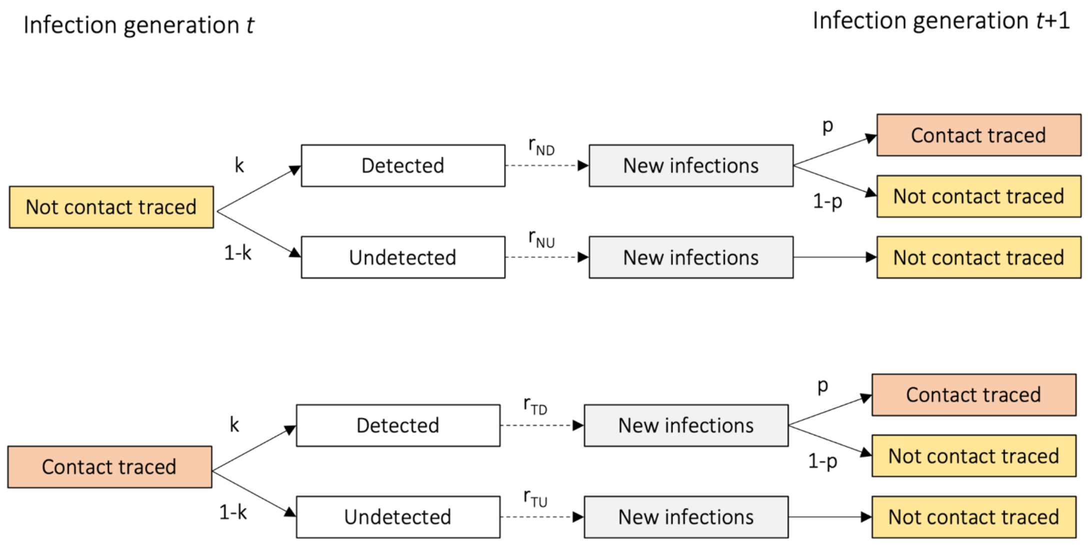

© 2023 Chitwood MH et al. *JAMA Network Open.*
