# Supplementary material for: Estimated Testing, Tracing, and Vaccination Targets for Containment of the US Mpox Outbreak
Source: JAMA Netw Open. 2023 Jan 13;6(1):e2250984. doi: 10.1001/jamanetworkopen.2022.50984 (PMC9857202; doi:10.1001/jamanetworkopen.2022.50984)
Supplement: Supplement 2. — Data Sharing Statement [file jamanetwopen-e2250984-s002.pdf]

Data Sharing Statement

Chitwood. Estimated Testing, Tracing, and Vaccination Targets for Containment of the US Mpox Outbreak. *JAMA Netw Open*. Published January 13, 2023.
doi:10.1001/jamanetworkopen.2022.50984

Data
Data available: Yes
Data types: Data (not involving human participants), Other (please specify)
Additional Information: Code to generate results, recreate figures
How to access data: https://github.com/mel-hc/contact_tracing/tree/pox_changes
When available: With publication

Supporting Documents
Document types: Statistical/analytic code
How to access documents: https://github.com/mel-hc/contact_tracing/tree/pox_changes
When available: With publication

Additional Information
Who can access the data: Anyone
Types of analyses: Any purpose
Mechanisms of data availability: Freely available on GitHub
